# Integrated transcriptomics, proteomics, and metabolomics analysis reveals newcastle disease virus reshapes glycerophospholipid metabolism

**DOI:** 10.1186/s12864-026-12760-5

**Published:** 2026-04-06

**Authors:** Yifan Sun, Tian Fang, Lei Tan, Cuiping Song, Xusheng Qiu, Ying Liao, Yingjie Sun, Xiufan Liu, Chan Ding, Chunchun Meng

**Affiliations:** 1 Shanghai Veterinary Research Institute, Chinese Academy of Agricultural Science, Shanghai, 200241 China; 2 Animal Infectious Disease Laboratory, College of Veterinary Medicine, Yangzhou University, Yangzhou, 225009 China; 3 School of Agriculture and Biology, Shanghai Jiao Tong University, Shanghai, 200240 China; 4 Laboratory of Veterinary Microbiology and Animal Infectious Diseases, College of Animal Sciences and Veterinary Medicine, Guangxi University, Nanning, 530004 Guangxi China; 5 Jiangsu Co-innovation Center for Prevention and Control of Important Animal Infectious Diseases and Zoonosis, Yangzhou University, Yangzhou, 225009 China; 6 Jiangsu Key Laboratory of Zoonosis, Yangzhou University, Yangzhou, 225009 China

**Keywords:** Newcastle disease virus, transcriptomics, proteomics, metabolomics, glycerophospholipid metabolism

## Abstract

**Background:**

Newcastle disease virus (NDV), a significant avian pathogen and promising oncolytic agent, relies on host metabolic pathways for replication. However, the metabolic alterations induced by NDV, particularly the connections at the gene and protein levels, remain poorly characterized.

**Results:**

This study employed integrated transcriptomic, proteomic, and non-targeted metabolomic analyses to delineate the global metabolic changes in NDV-infected A549 cells. We identified 8,101 differentially expressed genes (DEGs), 1,587 differentially expressed proteins (DEPs), and 257 differentially expressed metabolites (DEMs) associated with organelle function, innate immunity, and metabolism. Crucially, our multi-omics approach revealed that NDV significantly remodels glycerophospholipid metabolism. NDV depleted Lysophosphatidylcholine (LPC) and Lysophosphatidylethanolamine (LPE), as well as specific phosphatidylcholine (PC) and phosphatidylethanolamine (PE) species, while increasing phosphatidylserine (PS) at the late stage of infection. Strikingly, exogenous supplementation of unsaturated fatty acids, choline, phosphorylcholine, ethanolamine, phosphatidylethanolamine, and inositol markedly enhanced NDV replication. Concomitantly, NDV infection upregulated the transcriptional levels of key enzymes involved in glycerophospholipid biosynthesis.

**Conclusions:**

This study demonstrates for the first time that NDV actively reprograms host glycerophospholipid metabolism to facilitate viral replication. This study uncovers a novel mechanism of NDV-host interaction and provides crucial insights for oncolytic strategies targeting this metabolic vulnerability.

**Supplementary Information:**

The online version contains supplementary material available at 10.1186/s12864-026-12760-5.

## Introduction

Newcastle disease (ND), caused by the virulent strains of Newcastle disease virus (NDV), is a devastating, acute, and highly contagious disease posing a major threat to the global poultry industry [1–3]. NDV, classified within the genus *Orthoavulavirus* of the family *Paramyxoviridae*, is a non-segmented, negative-sense, single-stranded RNA virus [4, 5]. Beyond its veterinary significance, NDV has emerged as a promising platform for vaccine development (e.g., against influenza, SARS-CoV-2, HIV) [6–8]. In addition, NDV is also an oncolytic virus, which preferentially replicates in tumor cells and stimulates the body to produce anti-tumor immune response, making it a promising anti-tumor agent. Recent clinical advances, exemplified by a recombinant NDV carrying the α1,3GT gene (NDV-GT), have demonstrated remarkable efficacy in inducing hyperacute rejection in various refractory cancers [9].

Viruses, as obligate intracellular parasites, rely heavily on the host cellular material and energy supply for their replication [10, 11]. Although traditional single-omics technologies (such as transcriptomics or proteomics) can reveal host responses at specific levels, they are insufficient to comprehensively dissect the dynamic network through which viruses manipulate their hosts. Metabolomics is a robust approach for identifying the expression profiles of small molecule metabolites (such as amino acids, lipids, and sugars), and has been widely used in biological research involving animals, plants, and microorganisms [12–15]. Integrating metabolomics with transcriptomics and proteomics collectively offers a comprehensive cascade analysis spanning gene expression, protein function, and the resultant metabolic phenotype.

We previously reported metabolomic analyses both in vivo and in vitro, focusing on NDV infection in DF-1 cells and chicken plasma [16, 17]. These studies have expanded the understanding of the pathogenic mechanisms of NDV infection in avian species. Additionally, we have reported that in tumor cells, NDV promotes its own replication by upregulating SLC1A3 to regulate glutamine catabolism [18]. However, the specific alterations in lipid metabolism induced by NDV infection within tumor cells, particularly the connections at the gene and protein levels, remain largely unexplored.

To gain a holistic view of the metabolic reprogramming orchestrated by NDV in human tumor cells, this study employed an integrated transcriptomic, proteomic, and metabolomic approach to characterize the global changes in NDV-infected A549 cells. Transcriptomic and proteomic analyses revealed profound dysregulation not only in organelle function and innate immunity but also, crucially, in metabolic pathways. Metabolomic profiling specifically pinpointed significant perturbations in glycerophospholipid metabolism, characterized by the depletion of LPC and LPE, as well as specific PC and PE species, while increasing PS. Subsequent functional validation confirmed that glycerophospholipid metabolism promotes NDV replication. These findings significantly deepen our understanding of NDV oncolytic mechanisms and provide novel insights into virus-host metabolic interplay.

## Materials and methods

### Cells and virus

A549 and DF-1 cells were purchased from the American Type Culture Collection (Manassas, VA, USA). DF-1 cell line was cultured in Dulbecco’s modified Eagle’s medium (DMEM), and A549 cell line was cultured in Ham’s F-12 K supplemented with 10% foetal bovine serum (Excell Bio, Shanghai, China) at 37 °C in an atmosphere containing 5% CO_2_. A549 cells were used for all omics analyses and functional assays, while DF-1 cells were used only for virus titration.

NDV strain Herts/33 was obtained from the China Institute of Veterinary Drug Control (Beijing, China). Viral titers on DF-1 cells were determined as median tissue culture infective doses (TCID_50_) as described previously [19].

### Reagents and antibodies

Phosphorylcholine chloride (P302169), choline chloride (C108896), phosphatidylethanolamine (P303633), ethanolamine (E103802), and inositol (I108336) were purchased from Aladdin (Shanghai, China). L-Serine (HY-N0650), oleic acid (HY-N1446), linolenic acid (HY-W071746), palmitic acid (HY-N0830), and palmitoleic acid (HY-W011873) were purchased from MedChemExpress (Monmouth Junction, NJ, USA). Cell Counting Kit-8 (C0038) was purchased from Beyotime (Shanghai, China).

The following antibodies were used: anti-β-actin (AC026) from ABclonal Technology (Wuhan, China) and anti-NDV NP from our laboratory.

### Sample collection

A549 cells (10^7^ cells) were infected with NDV at a multiplicity of infection (MOI) of 3 at 37 °C. Following a 1 h absorption period, unattached viruses were removed and the cells were then washed three times with PBS and cultured in maintenance medium at 37 °C. At 24 h post-infection (hpi), the cells were washed three times with PBS and scraped with a cell scraper, then centrifuged at 500 g at 4 °C for 10 min. All samples were rapidly frozen in liquid nitrogen and stored at − 80 °C. For transcriptomic and proteomic analyses, three biological replicates (*N* = 3) were performed; for non-targeted metabolomic analysis, five biological replicates (*N* = 5) were performed.

### Transcriptomic sequencing and analysis

Transcriptomic analysis was performed on three biological replicates of the Mock group or the NDV group through RNA sequencing (RNA-seq). Total RNA was extracted using TRIzol according to the manufacturer’s instructions and treated with RNase-free DNase I (Thermo Fisher Scientific, MA, USA) to remove contaminating DNA. The quality of the extracted RNA was assessed using a NanoDrop 2000 spectrophotometer (Nanodrop Technologies, Wilmington, DE, USA). RNA integrity number was determined using the Agilent 2100 to evaluate the quality of the RNA. Total RNA was enriched for poly(A) RNA and fragmented for library construction. Construct the cDNA library using the Hieff NGS Ultima Dual-mode mRNA Library Prep Kit (Yeasen, Shanghai, China). The library sequencing was performed on the Illumina NovaSeq X Plus with a 2 × 150 paired-end configuration by Gene Denovo Biotechnology (Guangzhou, China).

To ensure data quality, FASTP is used for quality control of raw reads, filtering low quality data and obtaining clean reads [20]. On average, approximately 60 million reads were generated from each sample, with CleanData exceeding 99.5%. The reads were mapped to the Homo sapiens Genome Assembly GRCh38.p14 using HISAT2 (http://daehwankimlab.github.io/hisat2/) [21]. Mapping reads to the reference genome to obtain genome alignment of each sample with alignment rate was greater than 95%. For each transcription region, a fragment per kilobase of transcript per million mapped reads (FPKM) value was calculated to quantify its expression level and variations using StringTie v2.2.1 (https://ccb.jhu.edu/software/stringtie/) [22]. Differential RNA expression analysis was performed by DESeq2 v1.26.0 (https://bioconductor.org/packages/release/bioc/html/DESeq2.html) [23]. Genes for which the false discovery rate (FDR) < 0.05 and the absolute fold change (|FC|) > 2 were considered differentially expressed genes (DEGs). GO enrichment and KEGG pathway enrichment analyses of the DEGs were performed with R based on the hypergeometric distribution.

### DIA-MS proteomic analysis

Proteomics analysis was performed on three biological replicates of the Mock group or the NDV group through data-independent acquisition mass spectrometry (DIA-MS). The cells were lysed using lysis buffer (8 M Urea and 1% SDS). The concentration of the protein extract was determined by the BCA protein assay kit (Beyotime, Shanghai, China). Then, sequencing-grade trypsin (Promega, Madison, WI, USA) was used to digest the proteins into peptides. The desalted and lyophilized peptides were redissolved in 0.1% formic acid aqueous solution, followed by liquid chromatography-tandem mass spectrometry (LC-MS/MS) analysis. The entire system consisted of a timsTOF Pro2 mass spectrometer (Bruker Daltonics, Bremen, Germany) coupled in series with an UltiMate 3000 system (Thermo Fisher Scientific, MA, USA). The mass spectrometer operated in diaPASEF mode for DIA data acquisition by Gene Denovo Biotechnology (Guangzhou, China).

Peptides with FDR < 0.01 were quantified for proteins using the MaxLFQ algorithm. Proteins for which the FDR < 0.05 and |FC| > 1.5 were considered differentially expressed proteins (DEPs). GO enrichment and KEGG pathway enrichment analyses of the DEPs were performed with R based on the hypergeometric distribution.

### Non-targeted LC-MS metabolomic analysis

Non-targeted metabolomics analysis was performed on five biological replicates of the Mock and NDV-infected group through liquid chromatography-mass spectrometry (LC-MS). After the sample was slowly thawed at 4 °C, an appropriate amount of the sample was added to pre-cooled methanol/acetonitrile/water solution (2: 2: 1, v/v), vortexed, ultrasonicated at low temperature for 30 min, and allowed to stand at -20 °C for 10 min. The mixture was then centrifuged at 14,000 g at 4 °C for 20 min. The supernatant was collected and dried under vacuum. For mass spectrometry analysis, 100 µL of acetonitrile aqueous solution (acetonitrile: water = 1: 1, v/v) was added to reconstitute the sample, followed by vortexing and centrifugation at 14,000 g at 4 °C for 15 min. The supernatant was taken for analysis, and the Agilent 1290 Infinity LC ultra-high performance liquid chromatography system (UHPLC) (Agilent Technologies, Santa Clara, CA, USA) with a HILIC column was used for the analysis. The AB Triple TOF 6600 mass spectrometer (AB SCIEX, Framingham, MA, USA) was used to collect the primary and secondary spectra of the samples. The isolated metabolites were analyzed by mass spectrometry in both positive and negative ion modes by Gene Denovo Biotechnology (Guangzhou, China).

The mass spectrometry information was matched with the public metabolite databases HMDB (https://www.hmdb.ca/) and Metlin (https://metlin.scripps.edu/) to annotate the metabolites. PCA and OPLS-DA analyses were performed using ropls v1.6.2 (https://bioconductor.org/packages/release/bioc/html/ropls.html), and the stability of the models was evaluated using 7-fold cross-validation. Metabolites for which the p-value < 0.05, |FC| > 1, and variable importance in projection (VIP) values > 1 were considered differentially expressed metabolites (DEMs). KEGG pathway enrichment analyses of the DEMs were performed with R based on the hypergeometric distribution.

### SDS-PAGE and western blot analysis

Detailed steps for western blot analysis were as previously described [19]. In brief, total protein was extracted from the cells using RIPA lysis buffer (Beyotime) with protease and phosphatase inhibitors (Beyotime) on ice. The lysates were denatured and then subjected to SDS-PAGE. The resolved proteins were transferred to nitrocellulose membranes (Whatman, Little Chalfont, UK). The membranes were blocked and reacted with primary antibodies overnight at 4 °C and with horseradish peroxidase (HRP)-conjugated secondary antibodies for 1 h at room temperature. The immunoblot bands were visualized using ECL kits (Share-Bio, Shanghai, China).

### Quantitative real-time polymerase chain reaction

Detailed steps for reverse transcription quantitative polymerase chain reaction (RT-qPCR) were as previously described [19]. In brief, total RNA was extracted using TRIzol Reagent (Invitrogen, Carlsbad, CA, USA) according to the manufacturer’s instructions. cDNA was reverse transcribed from total RNA using Hiscript III Reverse Transcriptase (Vazyme Biotech, Nanjing, China) and oligo-dT primers. RT-qPCR was performed by adding cDNA, primers, and AceQ qPCR SYBR Green Master Mix (Vazyme Biotech). The ΔΔCT method was used to calculate the relative mRNA levels, which were normalized using β-actin mRNA. The primer sequences are shown in Table 1.

**Table 1:** Primers used for qPCR

Gene	Gene ID		Forward primers (5´–3´)	Reverse primers (5´–3´)
CT		5130	AGCGCCACCTCAGAAGATAAA	TGCACTTTGGAAGGAACCCC
CPT		56,994	ATTGCGCTCATTGGCAGACT	CCACCTAGAAATCCAAGAACTGG
ET		5833	CCATGATCCGGAACGGGC	CTGGTTGGAGTGGCCGTAAT
EPT		85,465	CTGGCTTTCTGCTGGTCGTA	GTTCTGCGAGCTTGCTTTCC
PGPS		9489	CGTGTTCTGGAGGCGACTG	TTCTGGACACAGGCAGCAAG
PSS1		9791	TCCAGCCTTATGGCGAATGG	CCCTTGTGGCGTATCGAAGA
PSS2		81,490	CCACACCTTAACCGTGCTCT	TGAGGTCTGGAAAATGGCCC
PIS		10,423	CCGGATTGTCTTCGCCATCA	GTTGACCAACAGGCACATGG

### Cell viability assay

Cell viability was assessed with the Cell Counting Kit-8 (CCK-8) according to the manufacturer’s protocol. Briefly, A549 cells were seeded in 96-well plates at 1 × 10⁵ cells/well in 100 µL of medium. Following an 18-hour treatment with different fatty acids and glycerophospholipid precursors, 10 µL of CCK-8 solution was added to each well, and the plates were incubated at 37 °C for 1 h. Absorbance was then measured at 450 nm to determine cell viability.

### Extracellular viral titer assay

To measure the viral titer in cell culture supernatants, a standard plaque assay was conducted [24]. DF-1 cells were plated in 12-well plates and grown overnight. The supernatant was serially diluted from 10^− 1^ to 10^− 6^ using serum-free DMEM. After washing the cells with PBS, 100 µL of each dilution was added per well and incubated for 2 h for viral adsorption. Unattached virus was then removed, and the cells were covered with a 2% carboxymethyl cellulose overlay. After 72 h, cells were fixed with 4% formaldehyde for 15 min and stained with 0.1% crystal violet for 15 min. Plates were rinsed with water, plaques were counted, and the viral titer was expressed as plaque-forming units per milliliter (PFU/mL).

### Statistical analysis

All data were presented as the mean ± standard deviation (SD) of at least three independent replicates. Western blot results were quantified using Image J software. Significant differences among groups were determined with a one-way analysis of variance using GraphPad Prism software. Differences were considered statistically significant when the value of P was less than 0.05.

## Results

### RNA sequencing and identification of differential expression genes (DEGs)

**Fig. 1:**
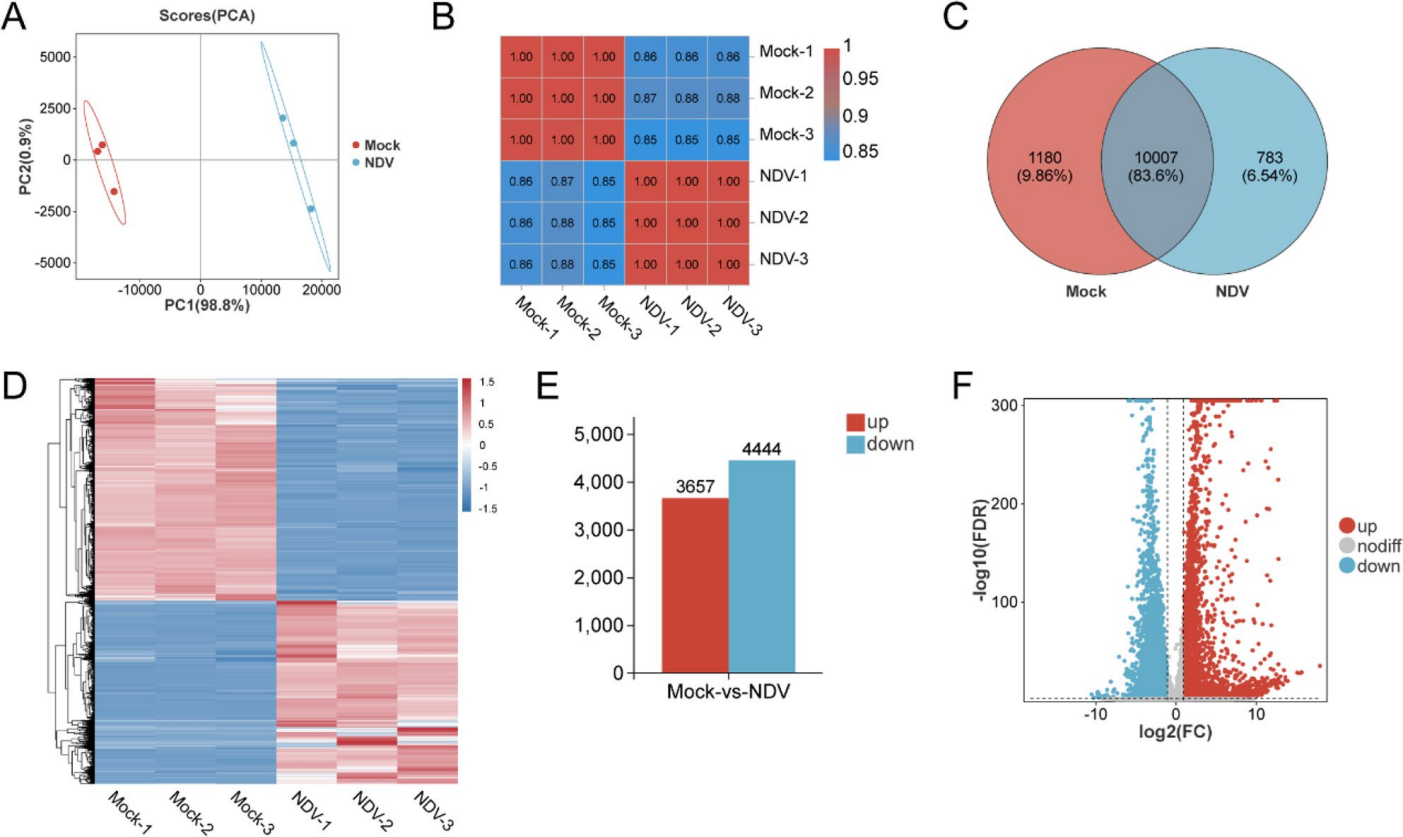
Analysis of transcriptomics data. A549 cells were infected with NDV at 3 MOI for 24 h and subjected to RNA-seq. **A** PCA score plot of Mock and NDV groups in the transcriptomics. **B** Pearson correlation between the groups in the transcriptomics. **C** Venn diagram of genes (FPKM > 1) between the groups in the transcriptomics. **D**-**F** Statistics of DEGs between the groups in the transcriptomics. Heatmap (**D**), bar chart (**E**), volcano plot (**F**). The threshold for DEGs was FDR < 0.05 and |FC| > 2. Colors in the heatmap represent the gene expression levels after Z-score normalization across different samples

To determine changes in gene expression upon NDV infection, RNA-seq analysis was conducted to investigate transcriptomics. Principal component analysis (PCA) revealed distinct clustering within the Mock group and the NDV group, with samples dispersed between groups, indicating high intra-group consistency and significant inter-group differences (Fig. 1A). The correlation analysis revealed a high degree of similarity between the replicate samples of the Mock group and the NDV group (Fig. 1B). In the Mock group, 11,187 genes were detected, while in the NDV group, 10,790 genes were detected (FPKM > 1). Among these, 10,007 genes were common to both groups, accounting for 83.6%. The Mock group had 1,180 unique genes, representing 9.86%, and the NDV group had 783 unique genes, representing 6.54% (Fig. 1C). Perform hierarchical clustering on the differential gene expression patterns and use a heatmap to visualize the clustering results (Fig. 1D). Compared to the Mock group, 8,101 differential expressed genes (DEGs) were identified in the NDV group, among which 3,657 genes were up-regulated and 4,444 genes were down-regulated (FDR < 0.05 and |FC| > 2) (Fig. 1E and F).

### DEGs function enrichment analysis

**Fig. 2:**
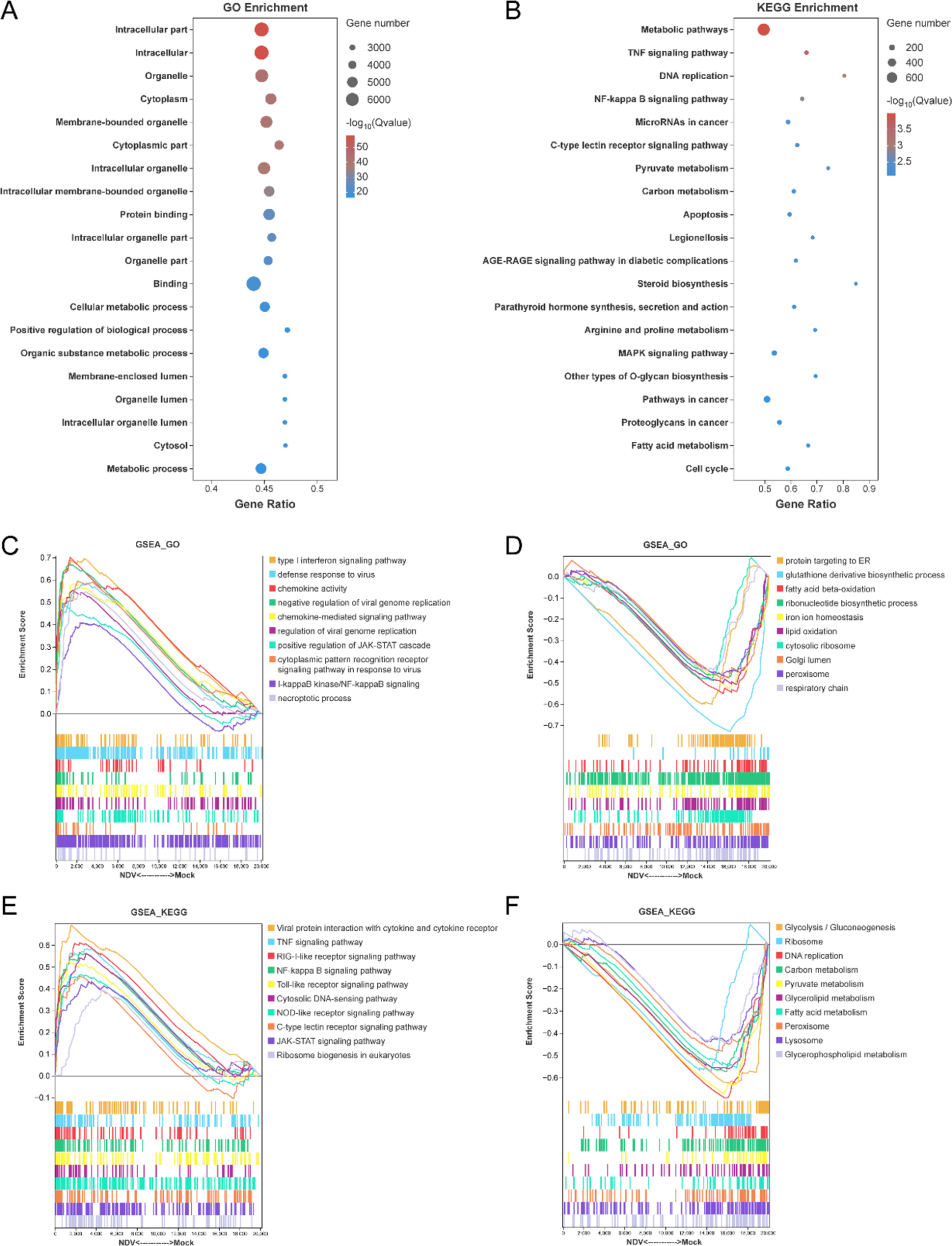
Enrichment analysis of DEGs. **A** The top20 of GO enrichment analysis of DEGs between the groups in the transcriptomics. **B** The top20 of KEGG enrichment analysis of DEGs between the groups in the transcriptomics. **C** GSEA analysis based on up-regulated GO terms in the transcriptomics. **D** GSEA analysis based on down-regulated GO terms in the transcriptomics. **E** GSEA analysis based on up-regulated KEGG terms in the transcriptomics. **F** GSEA analysis based on down-regulated KEGG terms in the transcriptomics

The GO enrichment analysis displays the top20 GO terms, including biological processes, cellular components, and molecular functions (Fig. 2A). DEGs are enriched in cellular components and biological processes, primarily including intracellular part, organelle, membrane-bounded organelle, as well as cellular metabolic process, positive regulation of biological process, and organic substance metabolic process. The KEGG enrichment analysis displays the top20 pathways, including TNF signaling pathway, DNA replication, NF-κB signaling pathway, pyruvate metabolism, carbon metabolism, and apoptosis (Fig. 2B). Gene Set Enrichment Analysis (GSEA) showed significantly differential GO terms and KEGG pathways following NDV infection. The upregulated GO terms included antiviral responses (type I interferon pathway, chemokine signaling, and NF-κB signaling), regulation of viral genome replication, and necrosis (Fig. 2C). The downregulated GO terms included organelles (endoplasmic reticulum, Golgi, ribosome, and peroxisome), and cellular metabolism (glutathione derivative synthesis and fatty acid beta-oxidation) (Fig. 2D). The upregulated KEGG pathways include pattern recognition receptor signaling (RIG-I-like receptor, Toll-like receptor, NOD-like receptor, and C-type lectin receptor) and innate immune signaling (TNF signaling, NF-κB signaling, and JAK-STAT signaling) (Fig. 2E). The downregulated KEGG pathways include organelles (ribosome, peroxisome, and lysosome) and metabolic pathways (pyruvate metabolism, glycerolipid metabolism, fatty acid metabolism, and glycerophospholipid metabolism) (Fig. 2F). These results from GO and KEGG enrichment analyses indicate that the DEGs following NDV infection are significantly involved in processes related to organelles, antiviral innate immunity, and cellular metabolism.

### Proteomics detection and identification of different expressed proteins (DEPs)

**Fig. 3:**
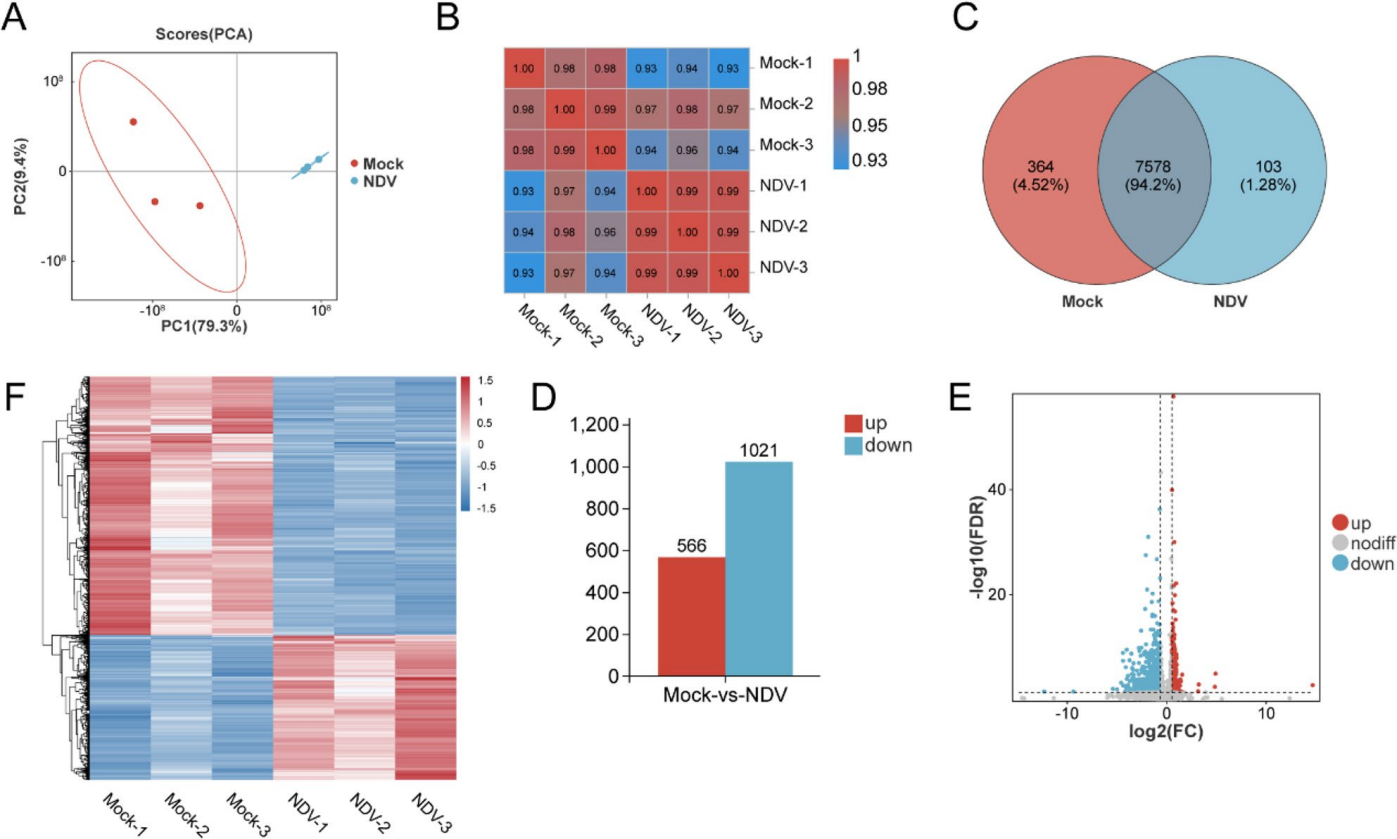
Analysis of proteomics data. A549 cells were infected with NDV at 3 MOI for 24 h and subjected to DIA-MS. **A** PCA score plot of Mock and NDV groups in the proteomics. **B** Pearson correlation between the groups in the proteomics. **C** Venn diagram of genes between the groups in the proteomics. **D**-**F** Statistics of DEPs between the groups in the proteomics. Heatmap (**D**), bar chart (**E**), volcano plot (**F**). The threshold for DEPs was FDR < 0.05 and |FC| > 1.5. Colors in the heatmap represent the gene expression levels after Z-score normalization across different samples

To determine changes in protein expression upon NDV infection, data-independent acquisition mass spectrometry (DIA-MS) analysis was conducted to investigate the proteomics. PCA revealed distinct clustering within the Mock group and the NDV group, with samples dispersed between groups, indicating high intra-group consistency and significant inter-group differences (Fig. 3A). The correlation analysis revealed a high degree of similarity between the replicate samples of the Mock group and the NDV group (Fig. 3B). In the Mock group, 7,942 proteins were detected, while in the NDV group, 7,681 proteins were detected. Among these, 7,578 proteins were common to both groups, accounting for 94.2%. The Mock group had 364 unique proteins, representing 4.52%, and the NDV group had 103 unique proteins, representing 1.28% (Fig. 3C). Perform hierarchical clustering on the differential protein expression patterns and use a heatmap to visualize the clustering results (Fig. 3D). Compared to the Mock group, 1,587 differential expressed proteins (DEPs) were identified in the NDV group, among which 566 proteins were up-regulated and 1,021 proteins were down-regulated (FDR < 0.05 and |FC| > 1.5) (Fig. 3E and F).

### DEPs function enrichment analysis

**Fig. 4:**
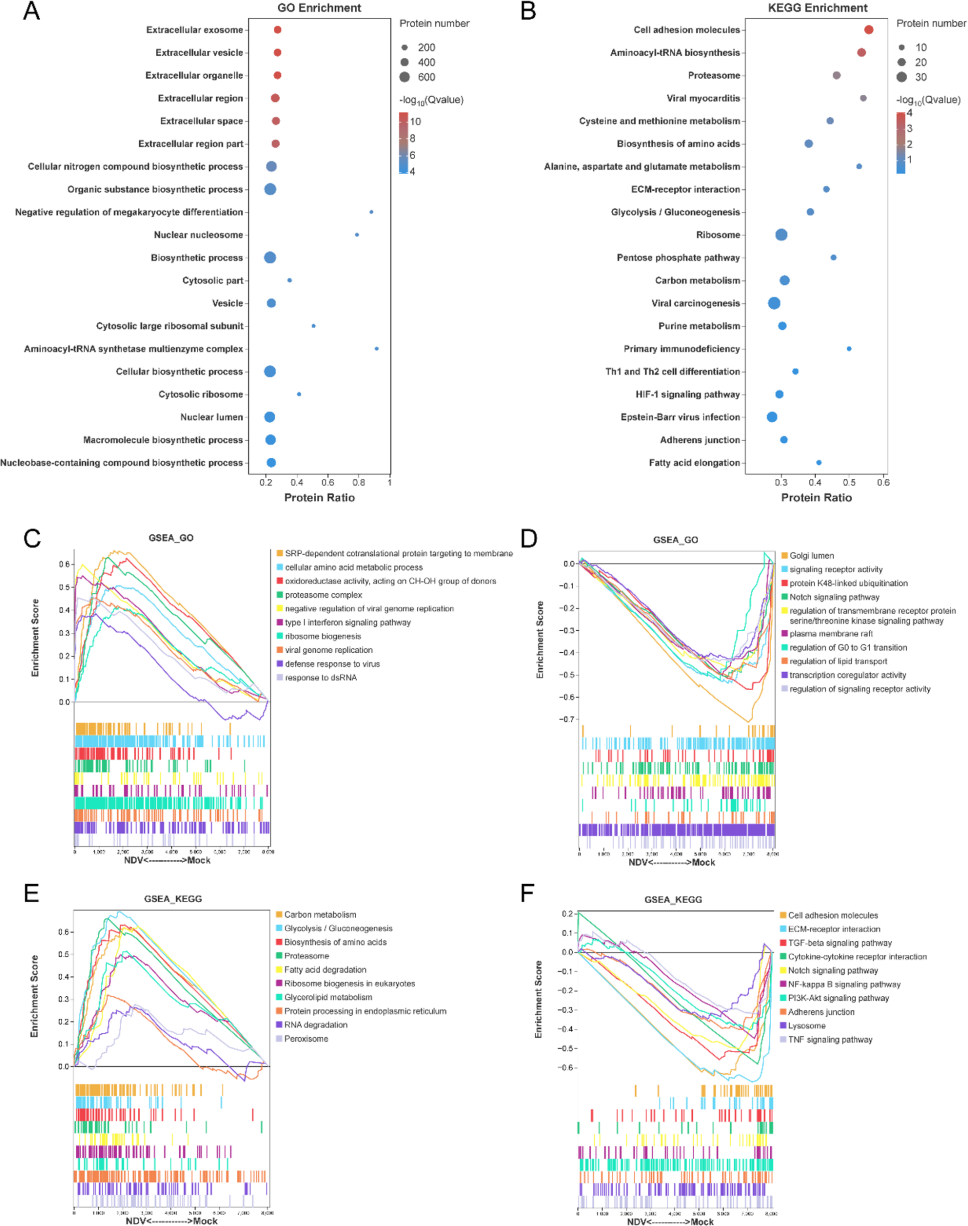
Enrichment analysis of DEPs. **A** The top20 of GO enrichment analysis of DEGs between the groups in the proteomics. **B** The top20 of KEGG enrichment analysis of DEGs between the groups in the proteomics. **C** GSEA analysis based on up-regulated GO terms in the proteomics. **D** GSEA analysis based on down-regulated GO terms in the proteomics. **E** GSEA analysis based on up-regulated KEGG terms in the proteomics. **F** GSEA analysis based on down-regulated KEGG terms in the proteomics

The GO enrichment analysis displays the top20 GO terms, including cellular components (extracellular region, cytosolic part, and nuclear nucleosome) and biological processes (cellular nitrogen compound biosynthetic process, organic substance biosynthetic process, and nucleobase-containing compound biosynthetic process) (Fig. 4A). The KEGG enrichment analysis displays the top20 pathways, including cell adhesion molecules, aminoacyl-tRNA biosynthesis, proteasome, and cysteine and methionine metabolism (Fig. 4B). GSEA showed significantly differential GO terms and KEGG pathways following NDV infection. The upregulated GO terms included antiviral responses (type I interferon pathway, response to dsRNA, and negative regulation of viral genome replication), viral genome replication, cellular amino acid metabolic process, and proteasome complex (Fig. 4C). The downregulated GO terms included Golgi lumen, signaling receptor activity, regulation of lipid transport, and cell cycle (Fig. 4D). The upregulated KEGG pathways are predominantly metabolic pathways, including carbon metabolism, glycolysis/gluconeogenesis, biosynthesis of amino acids, fatty acid degradation, and glycerolipid metabolism (Fig. 4E). The downregulated KEGG pathways include cell adhesion molecules, ECM-receptor interaction, TGF-beta signaling pathway, and cytokine-cytokine receptor interaction (Fig. 4F). These results from GO and KEGG enrichment analyses indicate that the DEPs following NDV infection are significantly involved in processes related to organelles, antiviral innate immunity, and cellular metabolism.

### The correlation analysis between transcriptomics and proteomics

**Fig. 5:**
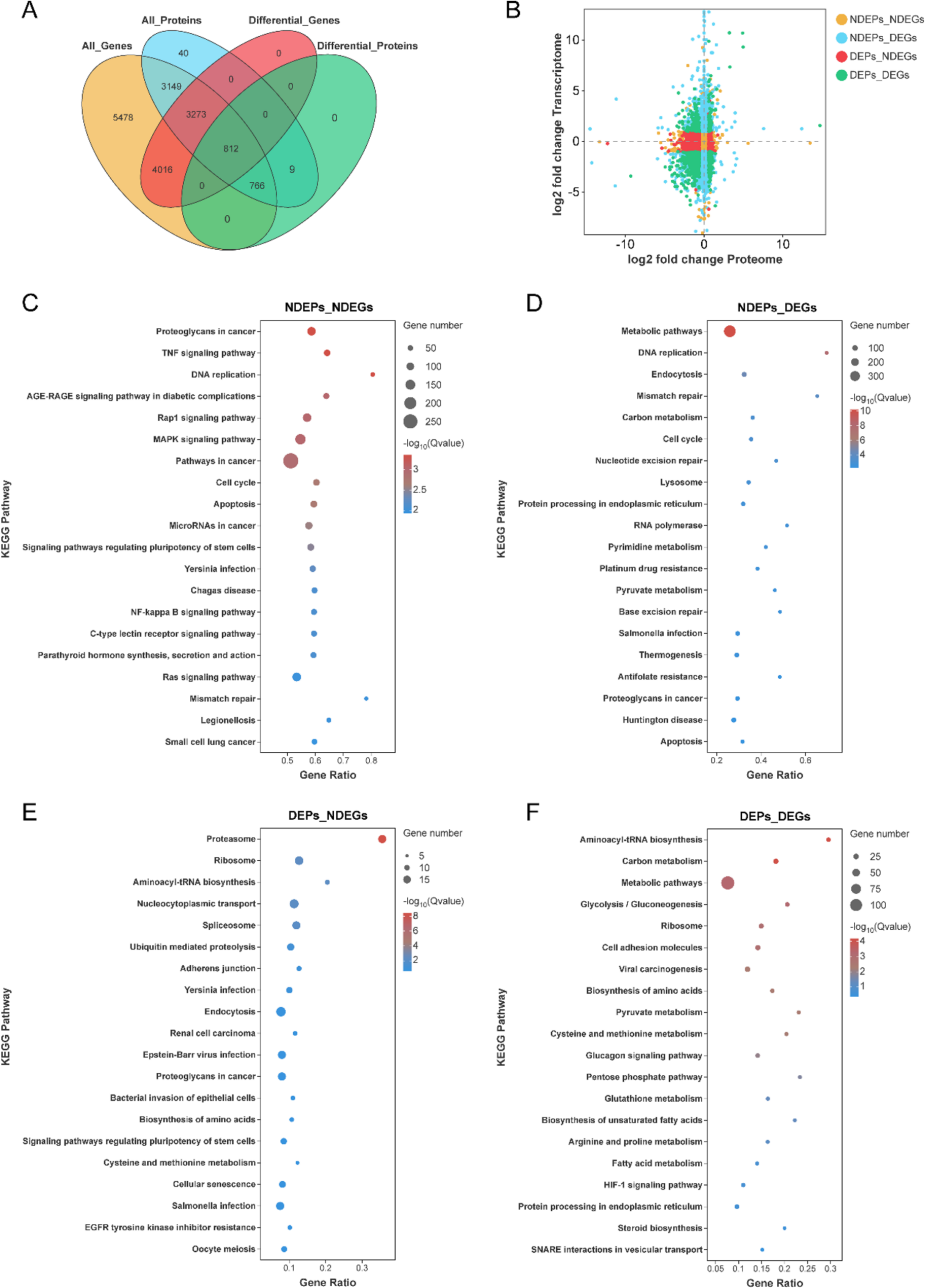
Combined analysis of transcriptomics and proteomics. **A** Venn diagram of the correlation numbers between transcriptomics and proteomics. The yellow color represents all genes and the blue color represents all proteins. The red color represents DEGs and the green color represents DEPs. **B** Comparison of expression ratios from transcriptomics (y-axis) and proteomics (x-axis) profiling based on quantitative proteins and the correlated genes in each pairwise. **C** The top20 of KEGG enrichment analysis of NDEPs_NDEGs group. **D** The top20 of KEGG enrichment analysis of NDEPs_DEGs group. **E** The top20 of KEGG enrichment analysis of DEPs_NDEGs group. **F **The top20 of KEGG enrichment analysis of DEPs_DEGs group

The total number of genes detected in the transcriptomics was 17,494, among which 8,101 genes showed significant differences (FDR < 0.05 and |FC| > 2), accounting for 46.3%. The total number of proteins detected in the proteomics was 8,049, among which 4,085 proteins showed significant differences (FDR < 0.05 and |FC| > 1.5), accounting for 50.8%. There were 812 genes that showed significant differences both at the mRNA level and the protein level (Fig. 5A). Among the 17,494 regulatory genes, only 8,000 in the study have corresponding proteins, which may be related to the sensitivity of transcriptomics and proteomics as well as post-transcriptional regulation [25, 26]. Based on the significance of changes, all genes were divided into four groups: non-differential expressed proteins (NDEPs)_non-differential expressed genes (NDEGs), NDEPs_DEGs, DEPs_NDEGs, and DEPs_DEGs (Fig. 5B). The KEGG enrichment analysis of NDEPs_NDEGs group included TNF signaling pathway, DNA replication, Rap1 signaling pathway, and MAPK signaling pathway (Fig. 5C). The KEGG enrichment analysis of NDEPs_DEGs group included Metabolic pathways, DNA replication, Endocytosis, and Mismatch repair (Fig. 5D). The KEGG enrichment analysis of DEPs_NDEGs group included proteasome, ribosome, aminoacyl-tRNA biosynthesis, and nucleocytoplasmic transport (Fig. 5E). The KEGG enrichment analysis of DEPs_DEGs group included aminoacyl-tRNA biosynthesis, carbon metabolism, metabolic pathways, and glycolysis/gluconeogenesis (Fig. 5F). The discrepancy between mRNA levels and protein levels may be attributed to several levels of regulation, such as RNA and protein turnover, post-translational modifications, protein conformational changes, and proteolysis.

### Non-targeted metabolomics detection and identification of differential metabolites (DEMs)

**Fig. 6:**
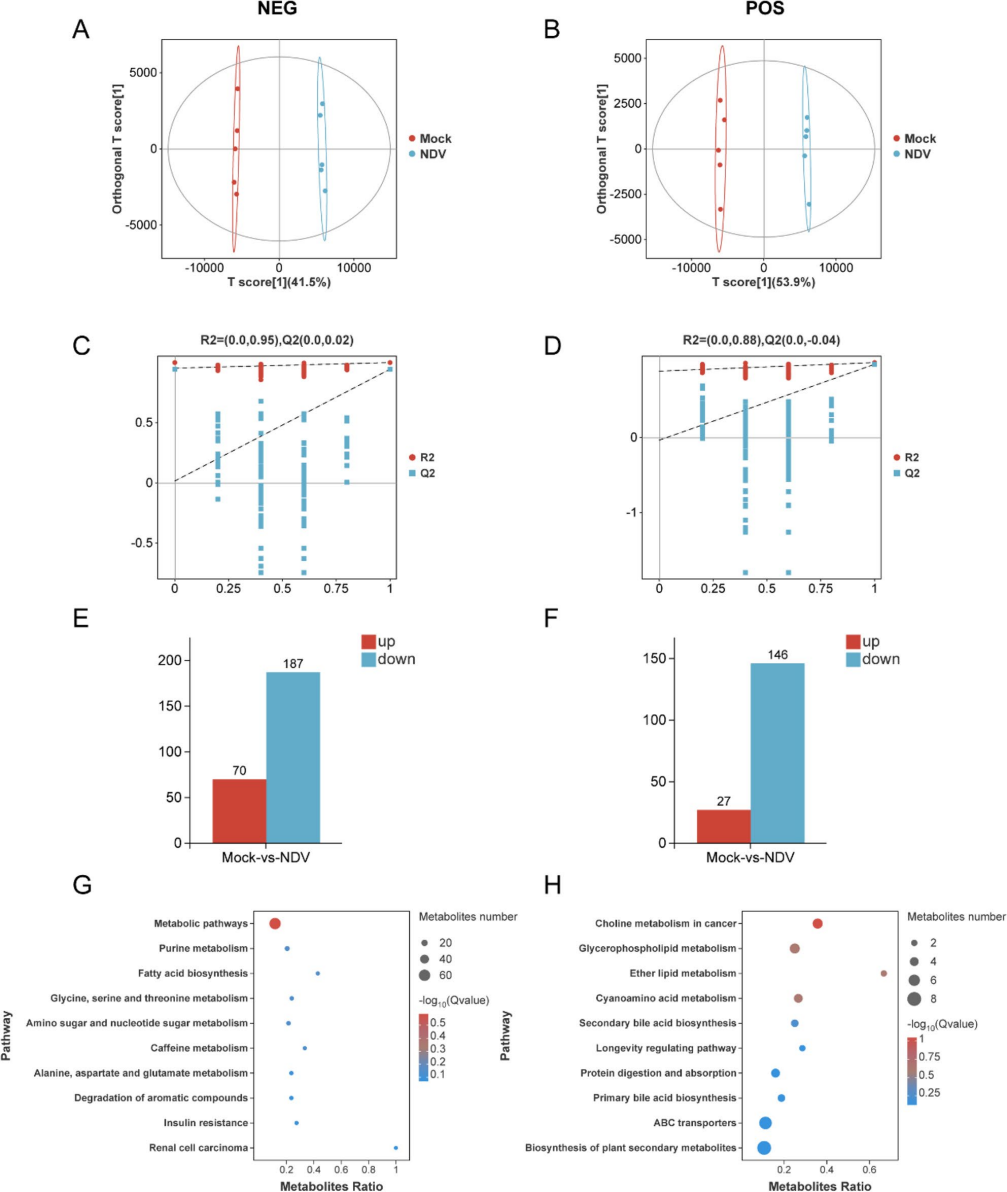
Analysis of non-targeted metabolomics data. A549 cells were infected with NDV at 3 MOI for 24 h and subjected to LC-MS. **A** OPLS-DA model analysis (NEG). **B** OPLS-DA model analysis (POS). **C** OPLS-DA model permutation test (NEG). **D** OPLS-DA model permutation test (POS). **E** Bar chart of DEMs (NEG). The threshold for DEMs was p-value < 0.05, |FC| > 1, and VIP > 1. **F** Bar chart of DEMs (POS). The threshold for DEMs was p-value < 0.05, |FC| > 1, and VIP > 1. **G** The top10 of KEGG enrichment analysis of DEMs (NEG). **H** The top10 of KEGG enrichment analysis of DEMs (POS)

To determine the changes in metabolites following NDV infection, LC-MS was employed to investigate the non-targeted metabolomics. LC-MS employed both positive ion mode (POS) and negative ion mode (NEG) to screen and identify a multitude of metabolites. To analyze the relationship between metabolite levels and sample groups, orthogonal partial least squares discriminant analysis (OPLS-DA) was employed. OPLS-DA model analysis revealed distinct clustering within the Mock group and the NDV group, with samples dispersed between groups, indicating high intra-group consistency and significant inter-group differences (Fig. 6A and B). Next, the plot of permutation test was performed to validate OPLS-DA reliability. The OPLS-DA model did not exhibit overfitting (R2Y > 0.85 and |Q2Y| < 0.05) (Fig. 6C and D). Compared to the Mock group, 257 (NEG) and 173 (POS) differentially expressed metabolites (DEMs) were identified in the NDV group (p-value < 0.05, |FC| > 1, and VIP > 1) (Fig. 6E and F). The KEGG enrichment analysis revealed that the top 10 pathways were predominantly associated with lipid metabolism, including fatty acid biosynthesis, glycerophospholipid metabolism, and ether lipid metabolism (Fig. 6G and H).

### NDV reshapes glycerophospholipid metabolism

**Fig. 7:**
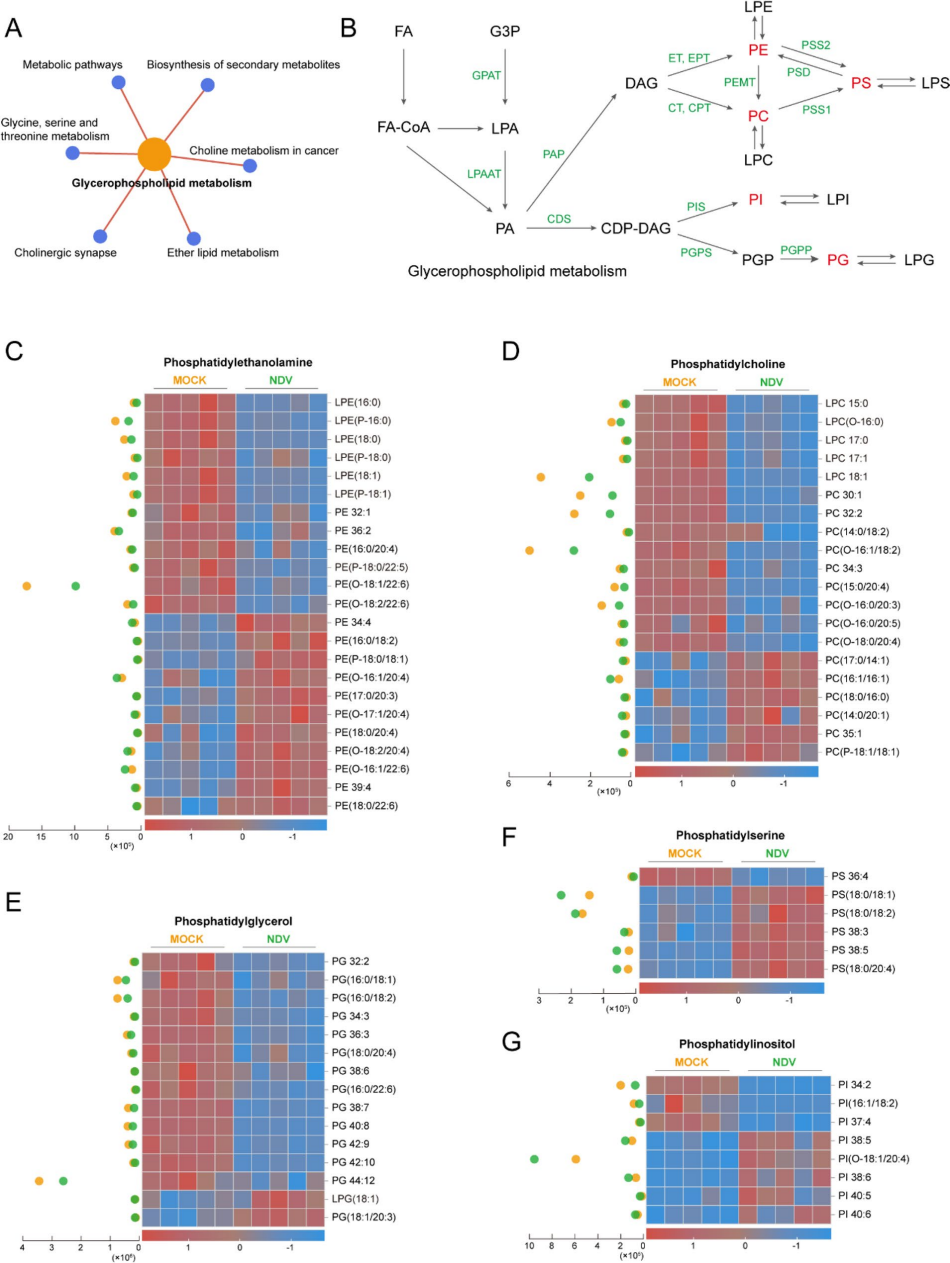
NDV reshapes glycerophospholipid metabolism. **A** KEGG pathway network centered on glycerophospholipid metabolism. **B** Glycerophospholipid metabolism pathway. Metabolites in black: FA, fatty acid; G3P, glycerol-3-phosphate; LPA, lysophosphatidic acid; PA, phosphatidic acid; DAG, diacylglycerol; CDP-DAG, CDP-diacylglycerol; PC, phosphatidylcholine; LPC, Lysophosphatidylcholine; PE, phosphatidylethanolamine; LPE, Lysophosphatidylethanolamine; PS, phosphatidylserine; LPS, Lysophosphatidylserine; PGP, phosphatidylglycerol phosphate; PG, phosphatidylglycerol; LPG, Lysophosphatidylglycerol; PI, phosphatidylinositol; LPI, Lysophosphatidylinositol. Enzymes in green: GPAT, glycerol-3-phosphate acyltransferase; LPAAT, lysophosphatidic acid acyltransferase; CDS, CDP-diacylglycerol synthase; PAP, phosphatidic acid phosphatase; ET, phosphoethanolamine cytidylyltransferase; EPT, diacylglycerol ethanolaminephosphotransferase; CT, phosphocholine cytidylyltransferase; CPT, diacylglycerol cholinephosphotransferase; PEMT, phosphatidylethanolamine N-methyltransferase; PSS1/2, phosphatidylserine synthase 1/2; PSD, phosphatidylserine decarboxylase; PIS, phosphatidylinositol synthase; PGPS, phosphatidylglycerophosphate synthase; PGPP, phosphatidylglycerophosphate phosphatase. **C**-**G** Heatmap analysis of glycerophospholipid metabolism final products between the groups. PE (**C**), PC (**D**), PG (**E**), PS (**F**), PI (**G**). The color of each rectangle represents the relative level of the differential metabolites. Red: upregulated; blue: downregulated

Generate a KEGG pathway network centered on glycerophospholipid metabolism, with multiple pathways associated, including biosynthesis of secondary metabolites, choline metabolism in cancer, ether lipid metabolism, cholinergic synapse, and glycine, serine and threonine metabolism (Fig. 7A). Glycerol 3-phosphate (G3P) and fatty acids (FA) are key precursors in glycerophospholipid metabolism, synthesizing the intermediate product phosphatidic acid (PA), and producing five final products: PC, PE, phosphatidylglycerol (PG), PS, and phosphatidylinositol (PI) (Fig. 7B). The carbon chain length of LPE varies between 16 and 18 carbons, existing in both saturated and monounsaturated forms, all of which decrease upon NDV infection (Fig. 7C). Notably, PE(O-18:1/22:6) exhibited the greatest abundance and fold change, showing a significant decrease upon NDV infection. Similarly, part PE increased upon NDV infection, but in absolute terms, the overall amount of PE shows a slight decrease. The carbon chain length of LPC varies between 15, 16, 17 and 18 carbons, existing in both saturated and monounsaturated forms, all of which decrease upon NDV infection (Fig. 7D). Specific PC species, such as PC 30:1, PC 32:2, PC(O-16:1/18:2), exhibited the greatest abundance and fold change, showing a significant decrease upon NDV infection. In absolute terms, the overall trend of PC was predominantly decreasing. Most PG species decreased upon NDV infection, among which PG 44:12 exhibited the greatest abundance and fold change (Fig. 7E). Most PI and PS species increased upon NDV infection, which presented a different change pattern compared to other final products (Fig. 7F and G). The above results indicate that NDV reshapes host glycerophospholipid metabolism.

### NDV replication depends on glycerophospholipid metabolism

**Fig. 8:**
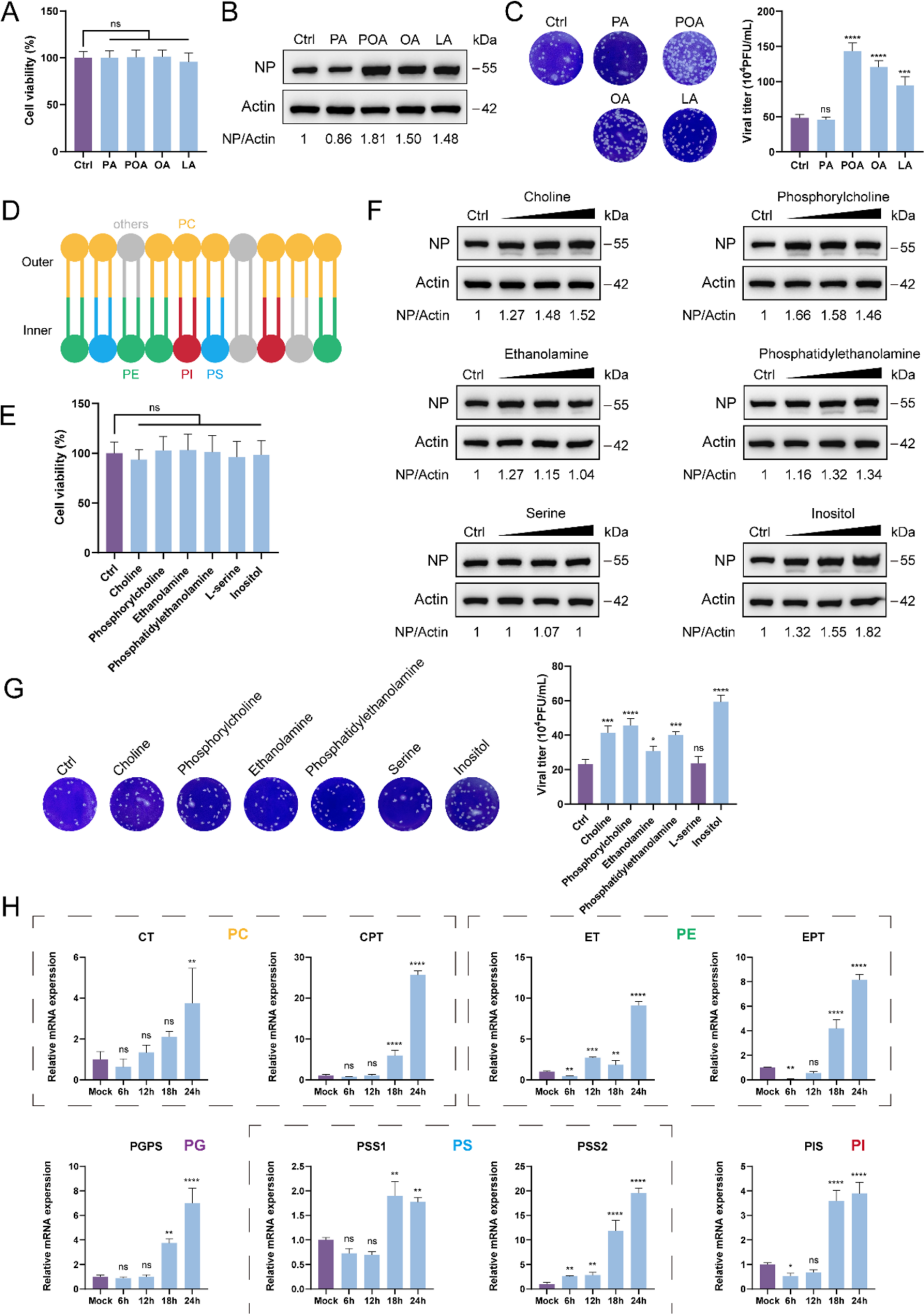
NDV replication depends on glycerophospholipid metabolism. **A** A549 cells were treated with PA, POA, OA, and LA (200 µM) for 18 h, and then cell viability was measured using CCK8 assay. **B**-**C** Effect of fatty acid saturation on NDV replication. A549 cells were infected with NDV at 0.01 MOI, supplemented with PA, POA, OA, and LA (200 µM). At 18 hpi, cells were collected to detect NP protein levels by western blot (**B**), and the supernatant was collected to measure progeny virus particles by plaque assay (**C**). **D** Schematic diagram of lipid distribution on the cell membrane. **E** A549 cells were treated with choline chloride (200 µM), phosphorylcholine chloride (200 µM), ethanolamine (2 mM), phosphatidylethanolamine (200 µg/mL), L-serine (2 mM), and inositol (2 mM) for 18 h, and then cell viability was measured using CCK8 assay. **F** A549 cells were infected with NDV at 0.01 MOI, supplemented with raw materials or intermediate products. Choline chloride (50, 100, 200 µM), Phosphorylcholine chloride (50, 100, 200 µM), Ethanolamine (0.5, 1, 2 mM), Phosphatidylethanolamine (50, 100, 200 µg/mL), L-serine (0.5, 1, 2 mM), Inositol (0.5, 1, 2 mM). At 18 hpi, cells were collected to detect NP protein levels by western blot. **G** A549 cells were infected with NDV at 0.01 MOI, supplemented with raw materials or intermediate products. Choline chloride (200 µM), Phosphorylcholine chloride (200 µM), Ethanolamine (2 mM), Phosphatidylethanolamine (200 µg/mL), L-serine (2 mM), Inositol (2 mM). At 18 hpi, the supernatant was collected to measure progeny virus particles by plaque assay. **H** Detecting the transcriptional levels of metabolic enzymes in glycerophospholipid metabolism. A549 cells were infected with NDV at 1 MOI and collected at 6, 12, 18, 24 h

To investigate the impact of fatty acid saturation on NDV replication, palmitic acid (PA, a saturated fatty acid), palmitoleic acid and oleic acid (POA and OA, monounsaturated fatty acids), and linoleic acid (LA, a polyunsaturated fatty acid) were separately added to the culture medium. Among them, PA and POA are 16 carbons, while OA and LA are 18 carbons. The cell viability results indicated that 200 µM fatty acids did not affect cell viability (Fig. 8A). Following the previous method, we examined the relationship between fatty acid saturation and NDV replication under low MOI [27]. The results of western blot and plaque assay indicate that saturated fatty acids do not affect NDV replication, whereas unsaturated fatty acids promote NDV replication (Fig. 8B and C).

The lipids on the cell membrane mainly include PC and PE, along with small amounts of PS, PI, and others (Fig. 8D). To investigate the roles of these four types of lipids in NDV budding, precursors of glycerophospholipid metabolism were added to the culture medium to observe the level of virus replication. The cell viability results indicated that specific concentration of precursors did not affect cell viability (Fig. 8E). The results of western blot and plaque assay indicate that exogenous addition of serine does not affect viral replication, whereas the addition of choline, phosphorylcholine, ethanolamine, phosphatidylethanolamine, and inositol all promote NDV replication (Fig. 8F and G). This suggests that PC, PE, and PI may play significant roles in the budding process of NDV.

To dynamically evaluate the synthesis of glycerophospholipid, the transcriptional levels of enzymes involved in glycerophospholipid metabolism were detected at different time points upon NDV infection. Among them, CT and CPT are involved in PC synthesis, ET and EPT in PE synthesis, PGPS in PG synthesis, PSS1 and PSS2 in PS synthesis, and PIS in PI synthesis. The results indicated that the transcriptional levels of these enzymes significantly increased during the middle and late stages of NDV infection (Fig. 8H). This suggests that NDV may upregulate the synthesis of glycerophospholipid to compensate for their consumption during viral budding.

## Discussion

This study presents the first integrated transcriptomic, proteomic, and metabolomic analysis of NDV-infected in human tumor cells (A549). Our multi-omics approach reveals that NDV extensively remodels host glycerophospholipid metabolism to facilitate viral replication. Collectively, we demonstrate that: (1) NDV infection induces global dysregulation encompassing organelle function, innate immunity, and metabolic pathways; (2) Glycerophospholipid metabolism is profoundly altered, characterized by depletion of specific PC and PE species; (3) Experimental validation confirms that exogenous supplementation of unsaturated fatty acids and key glycerophospholipid precursors (choline, phosphorylcholine, ethanolamine, phosphatidylethanolamine, inositol) significantly enhances NDV replication, likely through promoting viral budding. Although this study employed different infection doses and time points, and varying reaction conditions may lead to differences in response magnitude, the results demonstrate consistent trends. These findings establish, for the first time, that active reprogramming of host glycerophospholipid metabolism constitutes a novel mechanism underpinning NDV proliferation in tumor cells, revealing a critical metabolic target exploitable for antiviral or oncolytic strategies.

Integrated transcriptomic and proteomic analyses unveiled a profound NDV-induced disruption of cellular homeostasis, characterized by global organelle dysfunction and dysregulated innate immunity. Transcriptomic profiling revealed significant suppression of pathways associated with endoplasmic reticulum, Golgi apparatus, ribosome, and peroxisome functions, a finding corroborated at the protein level by the specific depletion of Golgi lumen components. Intriguingly, while transcriptional activation was observed for key innate immune sensors (e.g., RIG-I, TLRs) and downstream signaling cascades (e.g., NF-κB, JAK-STAT), proteomic analysis indicated a concurrent downregulation of critical effector proteins, such as cytokine receptors. This decoupling of upstream signaling activation from effector protein expression suggests a sophisticated viral strategy: NDV potentially subverts host defenses by blunting the output of activated immune pathways, while simultaneously exploiting the compromised organellar infrastructure for its own replication.

Fatty acids are categorized by chain length as short-chain (< 6 C), medium-chain (6–12 C), long-chain (14–20 C), and very long-chain (≥ 22 C) fatty acids [28, 29], or by saturation as saturated fatty acids (no double bonds), monounsaturated fatty acids (one double bond), and polyunsaturated fatty acids (two or more double bonds) [30, 31]. Typically, glycerophospholipids incorporate saturated fatty acids (predominantly C16:0 or C18:0) at the sn-1 position and unsaturated fatty acids (C18-C24) at the sn-2 position [32]. Consistent with this canonical distribution, our metabolomic analysis revealed similar fatty acid chain length patterns within the five major glycerophospholipids affected by NDV infection. Saturated fatty acids contain only carbon-carbon single bonds, which are more rigid and maintain the structure of the cell membrane; whereas unsaturated fatty acids contain carbon-carbon double bonds, which are more fluid and participate in vesicle formation and viral budding. Crucially, functional validation demonstrated that exogenous supplementation of unsaturated fatty acids, specifically POA (C16:1), OA (C18:1), and LA (C18:2), significantly enhanced NDV replication, whereas the saturated fatty acid PA (C16:0) had no effect. This differential impact likely stems from the critical role unsaturated fatty acids play in maintaining membrane fluidity. As an enveloped virus, NDV may depend on host membrane fluidity during both membrane fusion and budding. Membrane fluidity and curvature facilitating viral invasion and budding have been observed in various viruses [33–36].

Consistent with previous plasma metabolomics results, specific PC and PE species were depleted following infection with the virulent strain of NDV [17]. As enveloped viruses acquire host membrane lipids during budding [37, 38], the consumption of PC and PE, the most abundant glycerophospholipids in cellular membranes, likely reflects their incorporation into nascent viral envelopes. Supporting this notion, A lipid analysis of the SARS-CoV2 envelope revealed that PC constitutes the largest proportion at approximately 40–60%, which is similar to the PC composition in cell membranes [39]. Additionally, the most abundant fatty acids in the SARS-CoV2 envelope are 16:0, 18:0, and 18:1. This is similar to our result, where the most consumed fatty acid chains were 16:1 (e.g., PC(O-16:1/18:2)) and 18:1 (e.g., PE(O-18:1/22:6)). Currently, the lipid composition of the NDV envelope has not been elucidated. In addition, studies on flaviviruses have shown that different flaviviruses extensively deplete specific PC and PE species during late infection and peak viral replication [40, 41]. Similarly, lethal H1N1 influenza A virus (IAV) infection also exhibits depletion of specific PC and PE species [42, 43]. Therefore, we indirectly infer that the extensive budding of enveloped viruses depletes glycerophospholipids on the cell membrane, particularly specific PC and PE species, which is a widespread phenomenon.

Notably, PS was markedly upregulated among these five final products. PS, as a component on the inner side of the cell membrane, flips to the outer side in apoptotic cells infected by NDV. The exposed PS on viral envelopes can bind PS receptors (e.g., TIM-1) on target cells, facilitating viral attachment and entry [44–46]. Similar to NDV, IAV infection also increases PS on the cell membrane surface [42, 47]. Although exogenous addition of serine has no effect on viral replication, knockdown of the PS receptor significantly inhibits NDV replication (unpublished data), indicating that PS may play a role during the virus adsorption phase.

The mRNA levels of enzymes in glycerophospholipid metabolism were enhanced with the duration of viral infection. Notably, we observed a significant increase in phosphatidylserine synthase 2 (PSS2) mRNA levels, but not phosphatidylserine synthase 1(PSS1). It suggests the possible existence of a preferential conversion mechanism whereby the accumulated PS primarily originates from PE rather than PC. A study on flaviviruses has shown that different flaviviruses require distinct lipid remodeling enzymes, which are essential for efficient viral replication [40].

Exogenous supplementation of fatty acids and precursors suggests the importance of glycerophospholipid metabolism in NDV replication. Although the concentrations used had no effect on cell viability, they could not avoid influencing membrane properties, signaling pathways, and cellular stress responses. Additionally, this study has four limitations. First, our transcriptomic, proteomic, and metabolomic data were all obtained from the late stage of infection and thus cannot reflect the dynamic changes in glycerophospholipid metabolism during NDV infection. Although we demonstrated the importance of glycerophospholipid metabolism in NDV replication by exogenous addition of fatty acids and precursors, the dynamic changes in glycerophospholipid metabolism need to be further elucidated in subsequent studies. Second, we detected changes in the transcriptional levels of various metabolic enzymes involved in glycerophospholipid metabolism, but this does not indicate that the alterations in glycerophospholipids such as PC are caused by the upregulation of metabolic enzyme transcription. In the future, we will further validate the specific functions of these enzymes in NDV replication through methods such as gene knockout, inhibitor treatment, or enzyme activity assays. Third, the lipid composition of NDV viral particles remains unresolved. Based on the depletion patterns of host cell PC and PE, we hypothesize that NDV may incorporate them into the viral envelope. However, this assumption still requires validation through direct analysis of the lipid composition of purified viral particles. Fourth, this study primarily focuses on the metabolic regulatory mechanisms of NDV in tumor cells, but is limited to A549 cells, which requires validation in other tumor cell lines in the future. We also recommend conducting parallel verification in avian cells such as DF-1 and chick embryo fibroblasts (CEF) to enhance the universality of the findings.

## Conclusion

In conclusion, a multi-omics analysis combining transcriptomics, proteomics, and metabolomics was performed in NDV-infected tumor cells. In this study, 8,101 DEGs, 1,587 DEPs and 257 DEMs were identified, related to organelles, innate immunity and metabolism. Among them, NDV promotes its own replication by remodeling the host glycerophospholipid metabolism. Overall, this study provides multi-omics insights into NDV infection hosts and provides new insights into the oncolytic effects of NDV.

## Supplementary Information

Supplementary Material 1.

## Data Availability

The transcriptomics raw data are available in the National Genomics Data Center (NGDC) with the accession number HRA013907 (https://ngdc.cncb.ac.cn/gsa-human/browse/HRA013907). The proteomics raw data are available in iProX with the accession number IPX0014094001 (https://www.iprox.cn//page/subproject.html? id=IPX0014094001). The metabolomics raw data are available in Metabolights with the accession number MTBLS13267 (https://www.ebi.ac.uk/metabolights/editor/MTBLS13267).
