# Supplementary material for: Integrated transcriptomics, proteomics, and metabolomics analysis reveals newcastle disease virus reshapes glycerophospholipid metabolism
Source: BMC Genomics. 2026 Apr 6;27:546. doi: 10.1186/s12864-026-12760-5 (PMC13261996; doi:10.1186/s12864-026-12760-5)
Supplement: Supplementary file 1 — Supplementary Material 1. [file 12864_2026_12760_MOESM1_ESM.docx]

1. Original protein gel image in Figure 8A


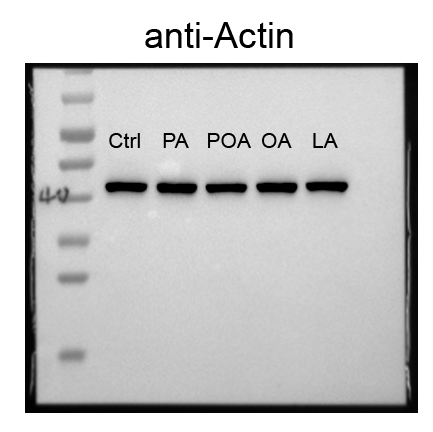

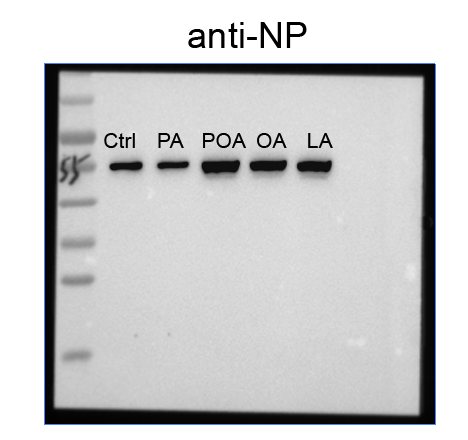


Effect of fatty acid saturation on NDV replication. NDV infected A549 cells at 0.01 MOI, supplemented with PA, POA, OA, and LA (200 µM). The level of NP protein was analyzed by Western blot after 18 hours of infection.

2. Original protein gel image of choline supplementation in Figure 8C


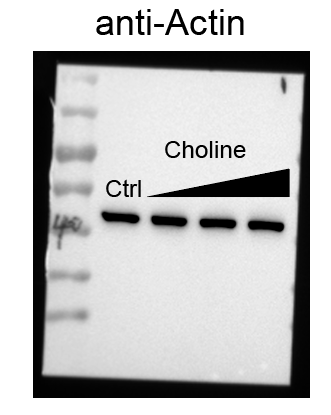

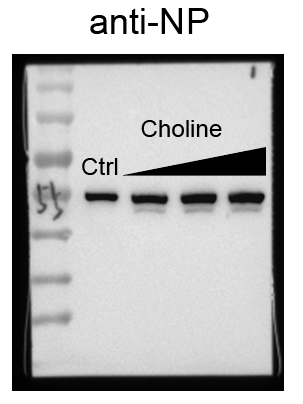


Effect of raw materials or intermediate products of glycerophospholipid metabolism on NDV replication. NDV infected A549 cells at 0.01 MOI, supplemented with choline chloride (50, 100, 200 µM). The level of NP protein was analyzed by Western blot after 18 hours of infection.

3. Original protein gel image of phosphorylcholine supplementation in Figure 8C


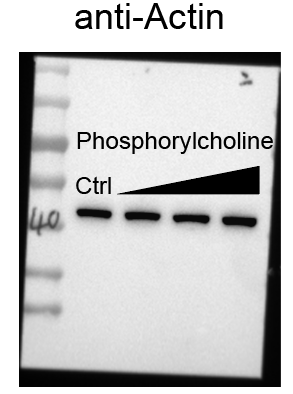

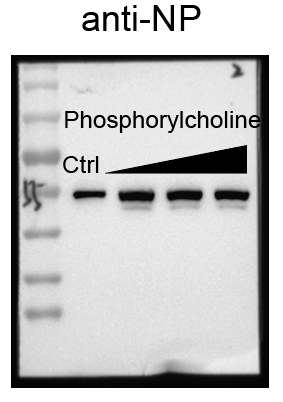


Effect of raw materials or intermediate products of glycerophospholipid metabolism on NDV replication. NDV infected A549 cells at 0.01 MOI, supplemented with phosphorylcholine chloride (50, 100, 200 µM). The level of NP protein was analyzed by Western blot after 18 hours of infection.

4. Original protein gel image of ethanolamine supplementation in Figure 8C


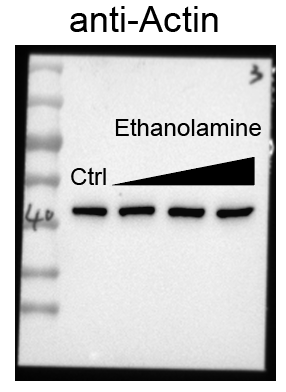

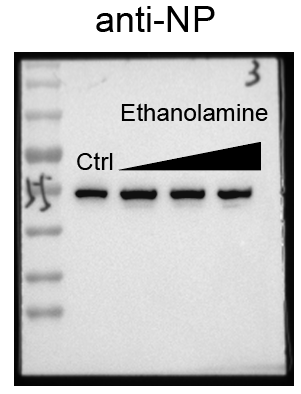


Effect of raw materials or intermediate products of glycerophospholipid metabolism on NDV replication. NDV infected A549 cells at 0.01 MOI, supplemented with ethanolamine (0.5, 1, 2 mM). The level of NP protein was analyzed by Western blot after 18 hours of infection.

5. Original protein gel image of phosphatidylethanolamine supplementation in Figure 8C


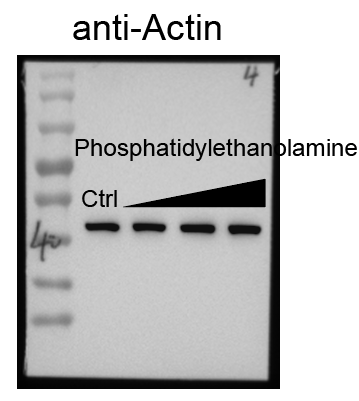

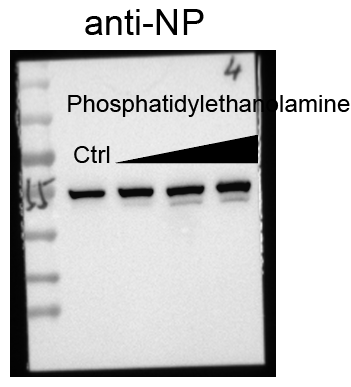


Effect of raw materials or intermediate products of glycerophospholipid metabolism on NDV replication. NDV infected A549 cells at 0.01 MOI, supplemented with phosphatidylethanolamine (50, 100, 200 µg/mL). The level of NP protein was analyzed by Western blot after 18 hours of infection.

6. Original protein gel image of serine supplementation in Figure 8C


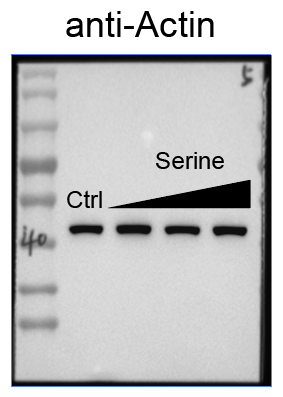

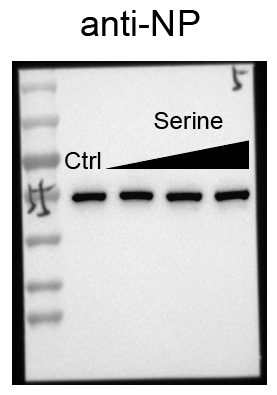


Effect of raw materials or intermediate products of glycerophospholipid metabolism on NDV replication. NDV infected A549 cells at 0.01 MOI, supplemented with L-serine (0.5, 1, 2 mM). The level of NP protein was analyzed by Western blot after 18 hours of infection.

7. Original protein gel image of inositol supplementation in Figure 8C


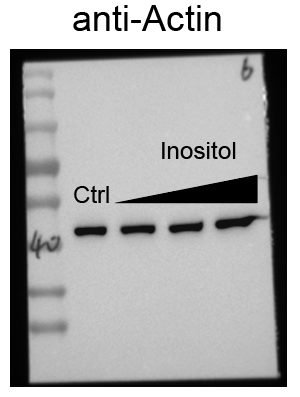

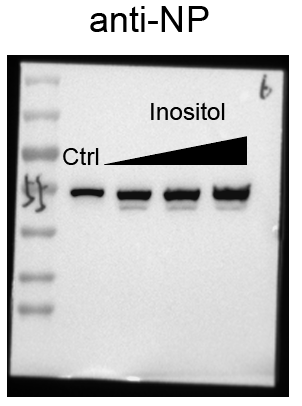


Effect of raw materials or intermediate products of glycerophospholipid metabolism on NDV replication. NDV infected A549 cells at 0.01 MOI, supplemented with inositol (0.5, 1, 2 mM). The level of NP protein was analyzed by Western blot after 18 hours of infection.
